# Diosgenin in polyendocrine metabolic ovarian syndrome: modulation of insulin resistance, inflammation, steroidogenesis, and metabolic dysfunction

**DOI:** 10.3389/fendo.2026.1906736

**Published:** 2026-09-16

**Authors:** Madhanraj Akilandeswari Pugalendhi, Devaraj Sankarganesh

**Affiliations:** School of Bio Sciences and Technology (SBST), Vellore Institute of Technology, Vellore, India

**Keywords:** adipocytes, obesity, diabetes, PCOS, PMOS, *PPARγ*, phytotherapy, HPO-axis

## Abstract

Polyendocrine Metabolic Ovarian Syndrome (PMOS) is a complex endocrinopathy with characteristic features of hyperandrogenism, ovulatory dysfunction, insulin resistance, and obesity, accompanied by other associated metabolic conditions, such as chronic inflammation, oxidative stress, and dyslipidemia. Currently available treatment options are metformin and thiazolidinediones, which address only the individual symptoms but associated with adverse side effects and variable outcomes. Therefore, the establishment of multi-targeted therapeutic advances remains elusive in managing the multifactorial complications of PMOS. Intriguingly, diosgenin, a steroidal sapogenin moiety abundantly present in *Dioscoreaceae* spp. has gained significant interest for its multimodal effects, including insulin sensitization and regulation of ovarian steroidogenesis, owing to its antioxidant, anti-inflammatory, and hypolipidemic properties. This review discusses the role of diosgenin in regulating the pathways, including PI3K/AKT, NF-κB, lipid homeostasis, and steroidogenesis, that are primarily disrupted in PMOS. In fact, a nuclear transcription factor, peroxisome proliferator-activated receptor gamma (*PPARγ*), acts as an upstream element of this vital regulatory machinery. Emerging evidence suggests that targeting *PPARγ* with diosgenin may improve insulin resistance, granulosa cell survival, and aromatase activity. This review presents the available preclinical and clinical evidence, as well as the challenges associated with the delivery, bioavailability, and future translational aspects of diosgenin in the management of PMOS. In summary, diosgenin may emerge as a multi-target candidate for the treatment of PMOS; however, its clinical translation requires mechanistic validation of *PPARγ*-mediated signaling, addressing inconsistencies in preclinical findings, optimizing bioavailability, and conducting well-designed clinical trials to establish its safety and therapeutic efficacy.

## 1 Introduction

Polycystic Ovary Syndrome (PCOS), recently renamed as Polyendocrine Metabolic Ovarian Syndrome (PMOS) to better reflect the multisystem endocrine and metabolic manifestations, is one of the most prevalent and mechanistically complex endocrine disorders that affects 10-13% of women at reproductive age (1, 2). WHO (2026) reported that 70% of PMOS cases remain undiagnosed. The clinical complications of PMOS include sporadic anovulation, excess androgen levels, and polycystic ovarian morphology (3). Moreover, it strongly contributes to other metabolic disorders, such as type II diabetes, obesity, cardiovascular disease, and non-alcoholic fatty liver disease (4). PMOS is also associated with depression and distress in women (5). Given the multifaceted clinical abnormalities of PMOS, several pharmacological interventions have been developed. Drugs, such as clomiphene citrate (6), metformin (7), letrozole (8), and thiazolidinediones, such as rosiglitazone (9) and pioglitazone (10), have primarily been used to ameliorate individual symptoms, such as anovulation/oligo-ovulation or insulin resistance, but not the multifactorial pathophysiology of PMOS. Moreover, some of these drugs are often idiosyncratic. For instance, metformin alleviates insulin resistance, but the response varies among patients. These notions underscore the importance of developing novel, efficient, and precise therapeutics for PMOS.

Mechanistically, complex interactions at the level of the hypothalamic-pituitary-ovarian (HPO) axis are crucial in PMOS, in that the pulse frequency of gonadotropin-releasing hormone (GnRH) determines the production of luteinizing hormone (LH) and follicle-stimulating hormone (FSH). As shown in **Figure 1**, under normal conditions, steroidogenesis in the ovary begins only when LH receptors are activated by LH, which is regulated by the physiological pulsatile frequency of GnRH. Subsequent synthesis of androgens (androstenedione and testosterone) occurs in the theca cells of the ovary in the presence of 17,20-lyase (CYP17A1) through the activation of the Cyclic Adenosine Monophosphate (cAMP)/Protein Kinase A (PKA) signaling pathway. Under the influence of FSH, these androgens then diffuse into the granulosa cells, where they are converted to estradiol (E2) via aromatization through the cAMP/PKA signaling pathway (11). In contrast, in PMOS, alterations in GnRH pulse frequency promote LH secretion, which stimulates the overproduction of androgen by ovarian theca cells, leading to hyperandrogenism, a hallmark of PMOS. Subsequently, FSH action is inhibited, aromatase (CYP19A1) activity is suppressed, and estrogen production is reduced in ovarian granulosa cells (12).

**Figure 1:**
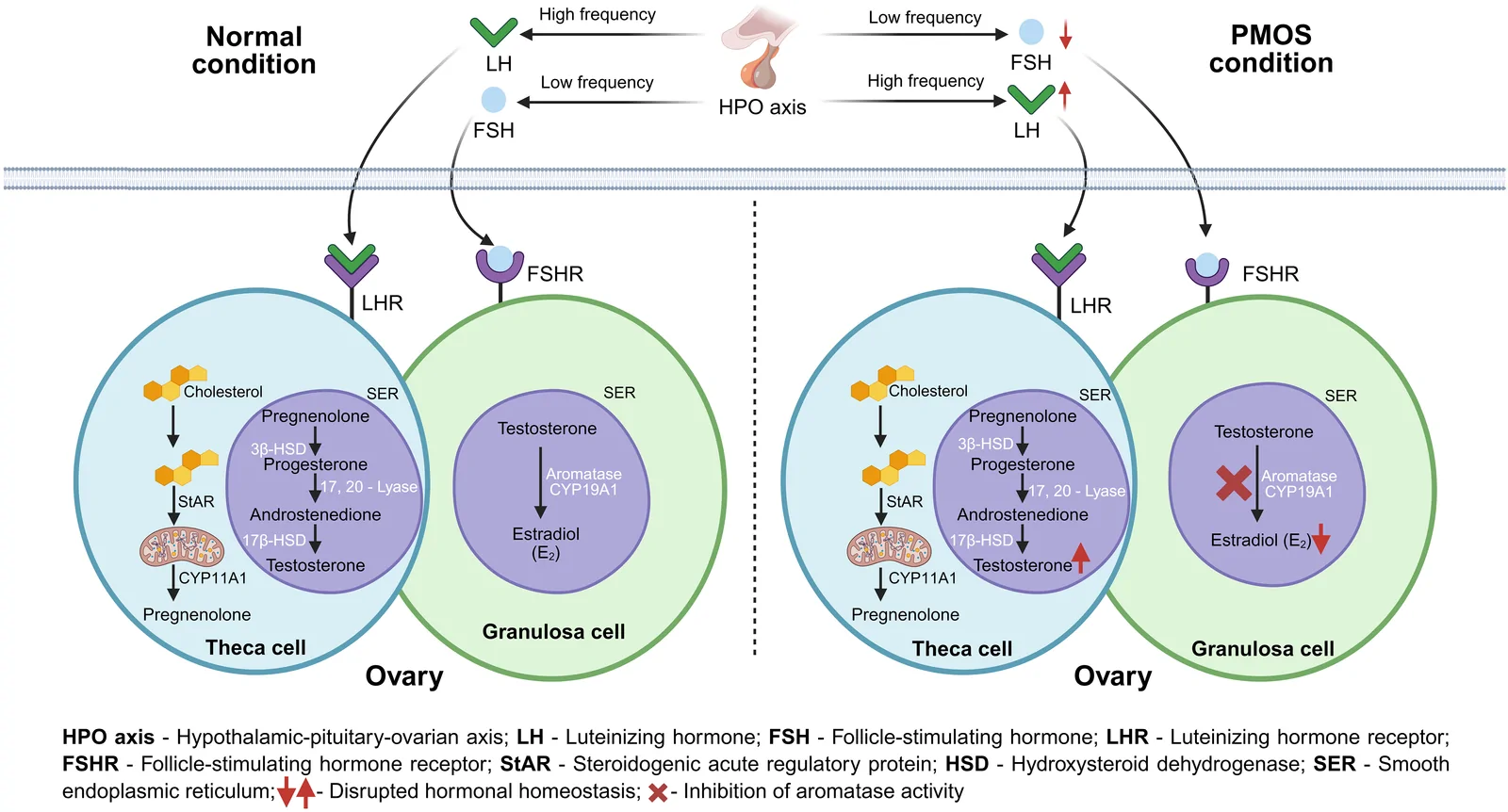
Normal ovarian steroidogenesis and altered hormonal regulation in PMOS showing increased androgen production and reduced CYP19A1-mediated estradiol synthesis.

In addition to hormonal dysregulation, molecular regulators, such as nuclear receptors, play vital roles in ovarian steroidogenesis. For instance, peroxisome proliferator-activated receptor gamma (*PPARγ*) is a key nuclear receptor that can modulate aromatase (13), along with retinoid X receptor (RXR), by forming a PPARγ-RXR complex (**Figure 2**). Aromatase is a key enzyme that converts androgens to estrogens in ovarian granulosa cells (14); therefore, modulating aromatase activity may partially restore steroidogenic imbalance. It has been shown that agonist-based activation of *PPARγ* upregulates aromatase expression in ovarian granulosa cells (15). In addition, thiazolidinediones (TZD), including rosiglitazone and pioglitazone, have been proven to activate *PPARγ* (16). However, TZDs have adverse side effects (17). At this juncture, identifying a viable alternative from natural sources that can modulate *PPARγ* activity is highly warranted.

**Figure 2:**
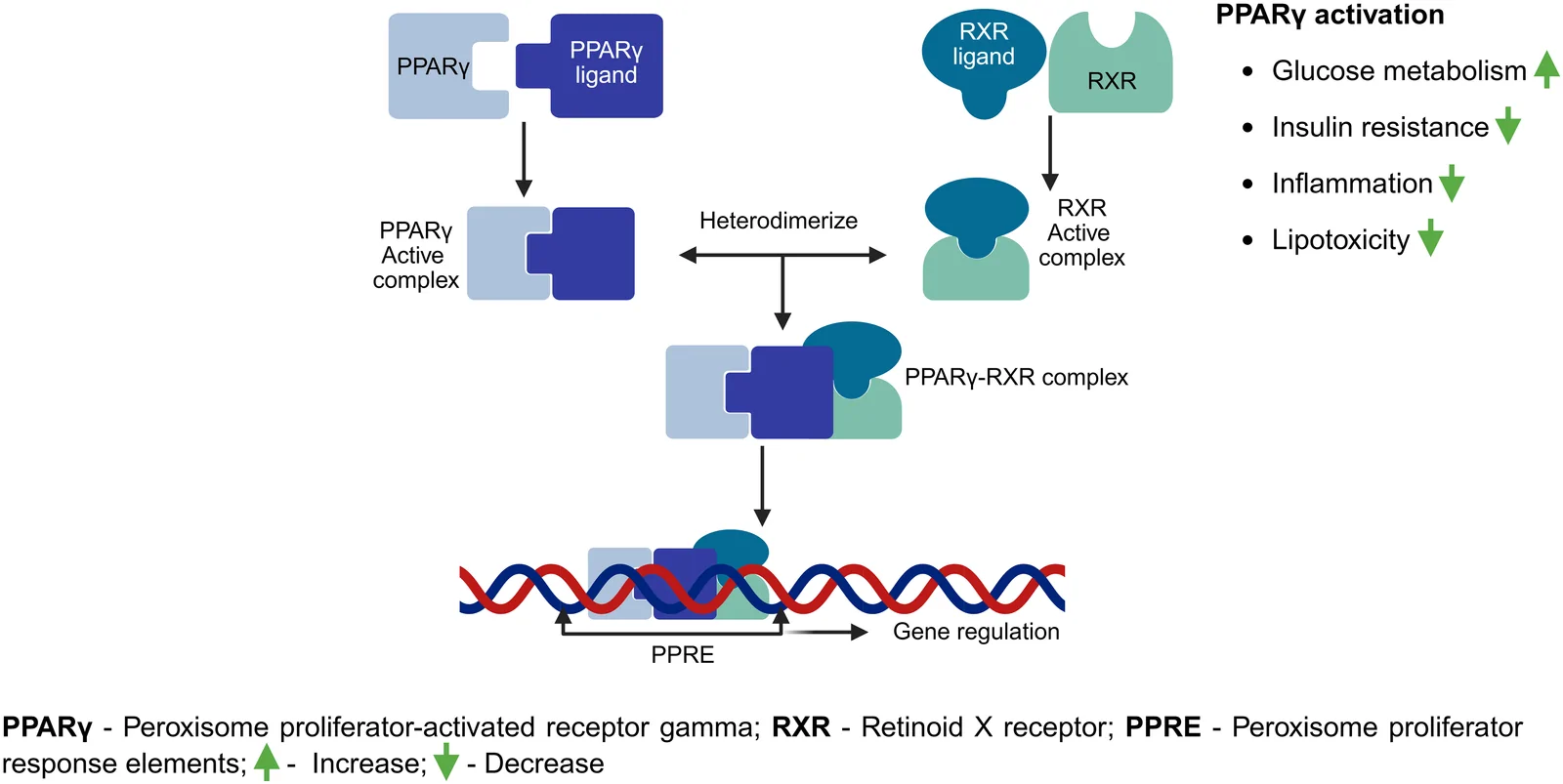
Ligand-mediated activation and transcriptional regulation by the PPARγ-RXR complex.

Not surprisingly, a plethora of studies have documented the roles of flavonoids, polyphenols, alkaloids (18), and phytoestrogens (19) in alleviating the symptoms of PMOS. Indeed, phytoestrogens are a promising category of molecules for PMOS treatment, owing to their affinity for estrogen-like effects and ability to act as selective estrogen receptor modulators (20). Notably, steroidal sapogenins, such as diosgenin, have gained particular interest in the management of PMOS and other associated metabolic conditions.

As depicted in **Figure 3**, diosgenin is a secondary metabolite in *Dioscoreaceae* spp. and a major compound of *Trigonella foenum-graecum* (Common name: fenugreek) and *Costus speciosus* (Common name: crepe ginger) (21). It has a high lipophilicity value (LogP=5.7) (22) and a steroidal backbone. Therefore, diosgenin is used as a precursor for many steroidal drugs. Pharmacologically, diosgenin has been proven to have protective effects on the cardiovascular and nervous systems. The literature also suggests that diosgenin may activate the secretion of lipid-soluble hormones (23), yet the evidence remains fragmentary. Therefore, we aimed to encapsulate the proven and possible therapeutic effects of diosgenin. This review provides evidence of the known mechanistic actions of diosgenin and highlights unexplored facets of its role in the management of PMOS.

**Figure 3:**
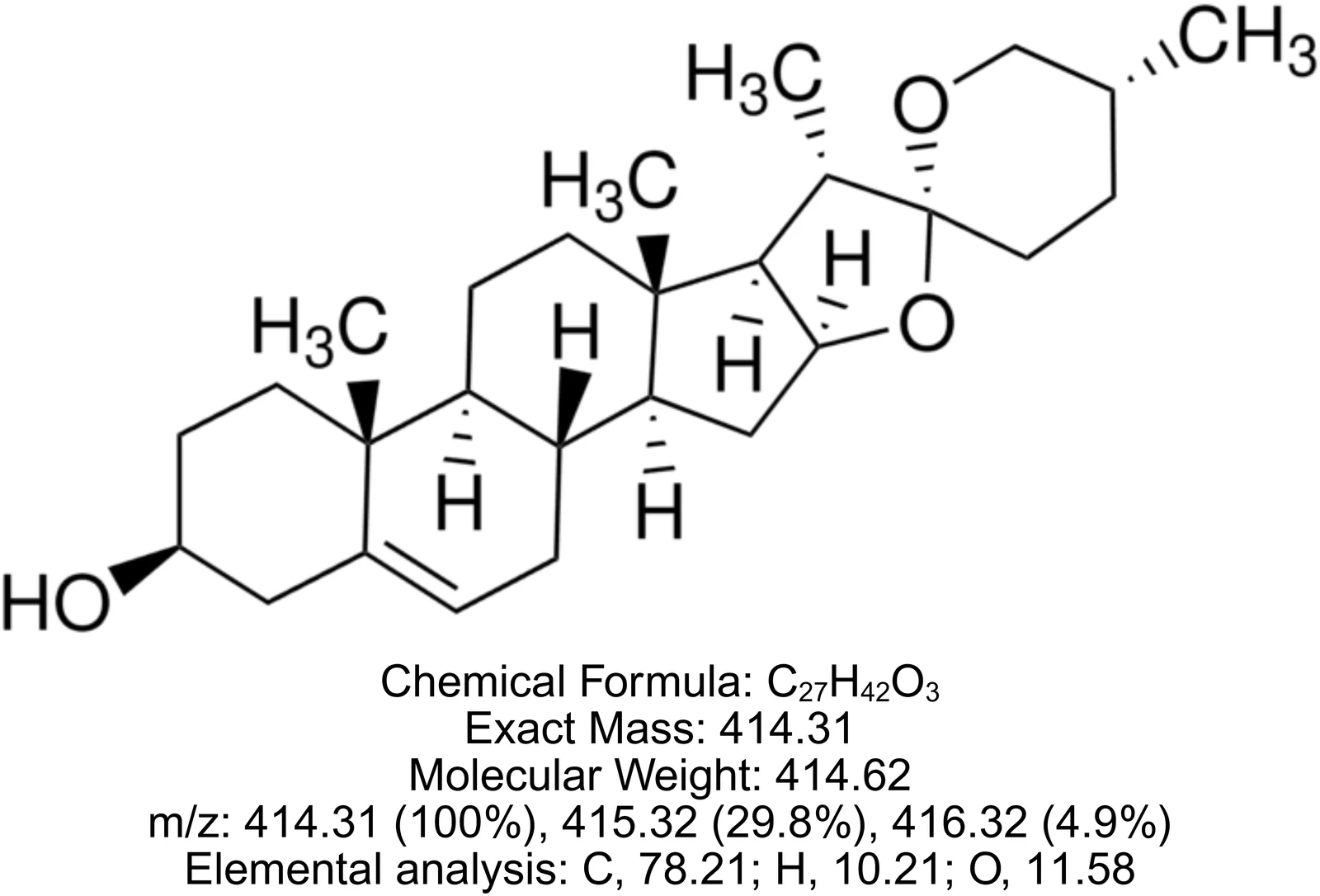
Chemical structure of diosgenin.

## 2 PMOS pathophysiology

The most deleterious clinical feature of PMOS is hyperandrogenism (HA), which is mainly due to the overproduction of androgens in ovarian theca cells (24). Androgens contributing to hyperandrogenic conditions include testosterone, androstenedione (A4), dehydroepiandrosterone sulfate (DHEAS), while sex hormone-binding globulin (SHBG), a glycoprotein, regulates the bioavailability of circulating sex hormones (25). As shown in **Figure 4**, hypersecretion of androgens, in the adrenal glands also exacerbates HA in PMOS (26). This, in turn, hampers glucose metabolism, alters the action of peripheral and intrahepatic insulin, and increases the release of free fatty acids (FFAs), thereby resulting in insulin resistance (IR) and other metabolic comorbidities (27). Indeed, the state of HA results in ovarian inflammation, abnormal apoptosis, and autophagy, leading to follicular impairment. Specifically, HA elevates endoplasmic reticulum stress in the ovarian microenvironment and triggers activation of nuclear factor kappa-light-chain-enhancer of activated B cells (NF-κB) and inflammasome signaling, ultimately leading to ovarian inflammation (28). Furthermore, a recent study provided molecular evidence that hypermethylation of *PPARγ1* is involved in HA and promotes insulin resistance in patients with PMOS (29).

**Figure 4:**
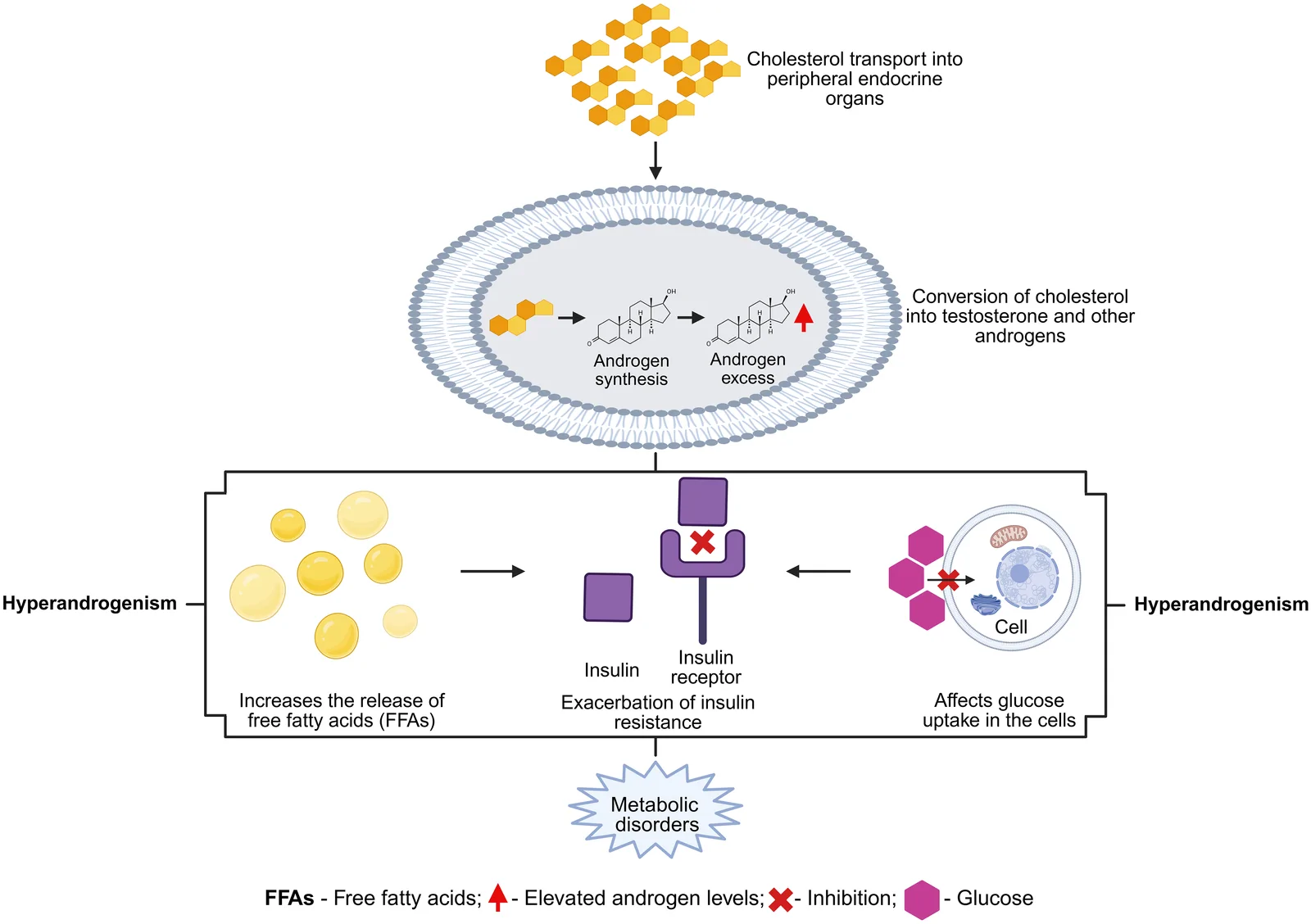
Hyperandrogenism-induced dysregulation of lipid metabolism and insulin signaling contributes to metabolic disorders in PMOS.

Hyperinsulinemia, which aggravates IR, is another key pathological feature of PMOS, observed in the ovaries and endometrium of patients (30). IR represents a central pathway linking reproductive anomalies and metabolic complications in PMOS (31). As depicted in **Figure 5**, it affects the HPO axis, which in turn leads to hypersecretion of adrenal cortisol and androgens, eventually hindering normal steroidogenesis in the ovaries. This hypersecretion from the adrenal gland inhibits the aromatization of androgens, which directly hampers the production of FSH and the maturation of primordial follicles. Subsequently, an increase in LH secretion and a decrease in estrogen and SHBG levels lead to amenorrhea and/or oligomenorrhea (32). These ovulatory issues are highly prevalent in obese PMOS patients due to increased IR. Although IR and obesity are closely associated, obesity is not a prerequisite for the development of PMOS, as IR is also observed in lean PMOS patients (33). This suggests that metabolic abnormalities arising from insulin impairment are not specifically dependent on obesity or increased adiposity (34, 35).

**Figure 5:**
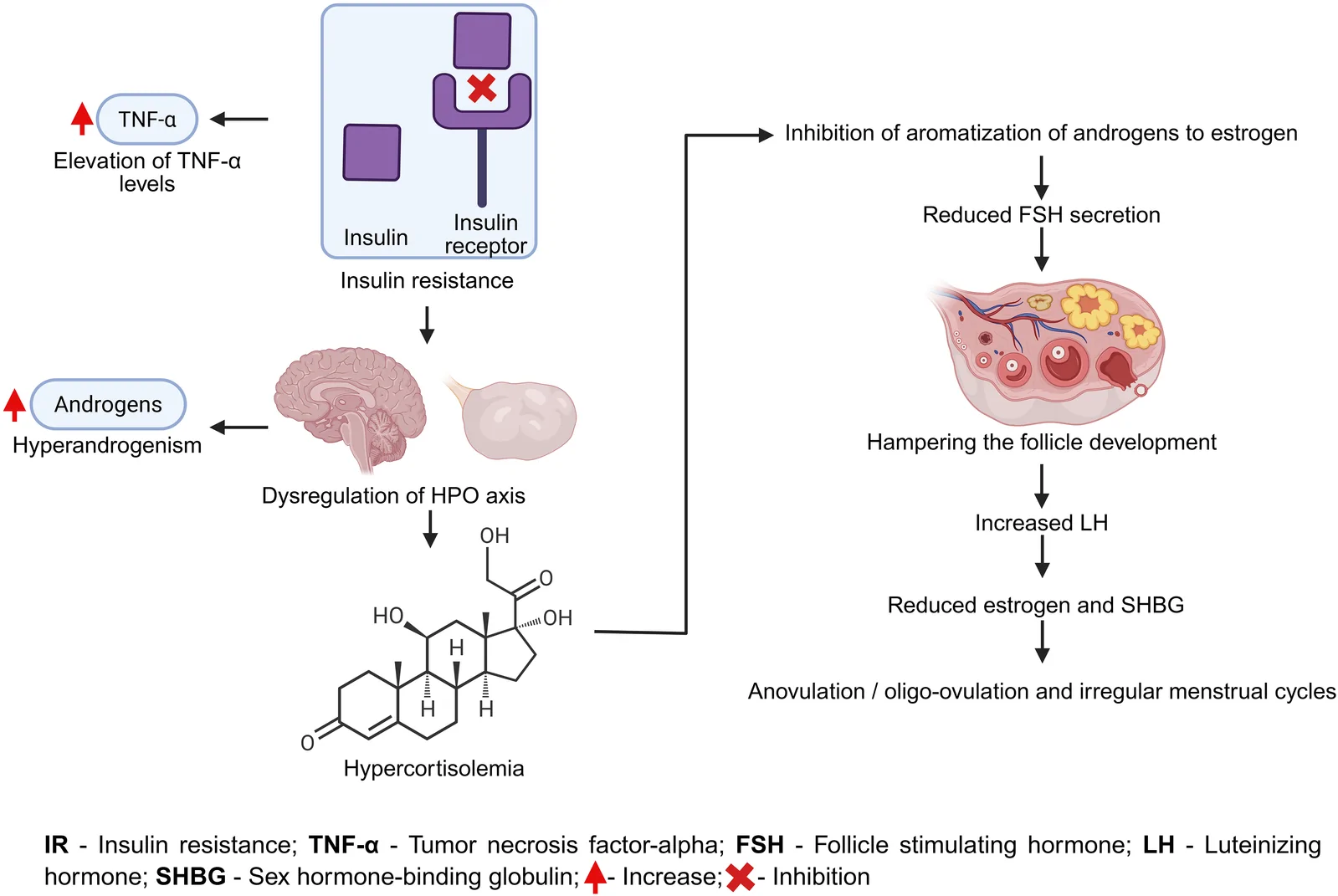
Role of insulin resistance in hormonal imbalance, follicular dysfunction, and menstrual irregularities associated with PMOS.

Chronic IR promotes a proinflammatory state, contributing to PMOS progression. Importantly, elevated levels of tumor necrosis factor-alpha (TNF-α) in obese PMOS patients suggest that PMOS is a chronic low-grade inflammatory condition (36). In addition, inflammatory markers, including TNF-α, are known to induce inflammation and apoptosis in renal cells (37). In patients with PMOS, C-reactive protein (CRP) levels are also increased, together with the hypersecretion of pro-inflammatory cytokines (38) and the elevated neutrophil-to-lymphocyte ratio (NLR) (39). The elevated NLR was also prevalent in patients with diabetes (40). IR can contribute to abnormal deposition of visceral adipose tissue, resulting in metabolic and hormonal disturbances associated with PMOS by increasing the release of FFAs and pro-inflammatory cytokines (41), leading to conditions such as diabetes, hypertension, cardiovascular risks, and inflammatory disorders (42). Adipose-derived secretory molecules, such as adiponectin, leptin, omentin, apelin, chimerin, irisin, resistin, vaspin, and visfatin play key roles in metabolic homeostasis (**Figure 6**). Dysregulation of these adipokines is considered a key pathological feature of PMOS (43). For instance, impaired leptin signaling in PMOS contributes to IR and other metabolic issues. Chimerin suppresses FSH-stimulated estrogen synthesis in immature rat granulosa cells at higher doses and decreases the mRNA and protein expression of aromatase (44), suggesting its involvement in the regulation of FSH-mediated follicular development (45). Visfatin, an important adipokine, has been reported to modulate insulin signaling through phosphorylation of insulin receptor and the activation of the phosphoinositide 3-kinase (PI3K), protein kinase B (AKT), and mitogen-activated protein kinase (MAPK) pathways, thereby contributing to the improvement of glucose homeostasis (46). Similarly, omentin improves insulin sensitivity by enhancing insulin-stimulated AKT phosphorylation and glucose uptake in adipocytes (47).

**Figure 6:**
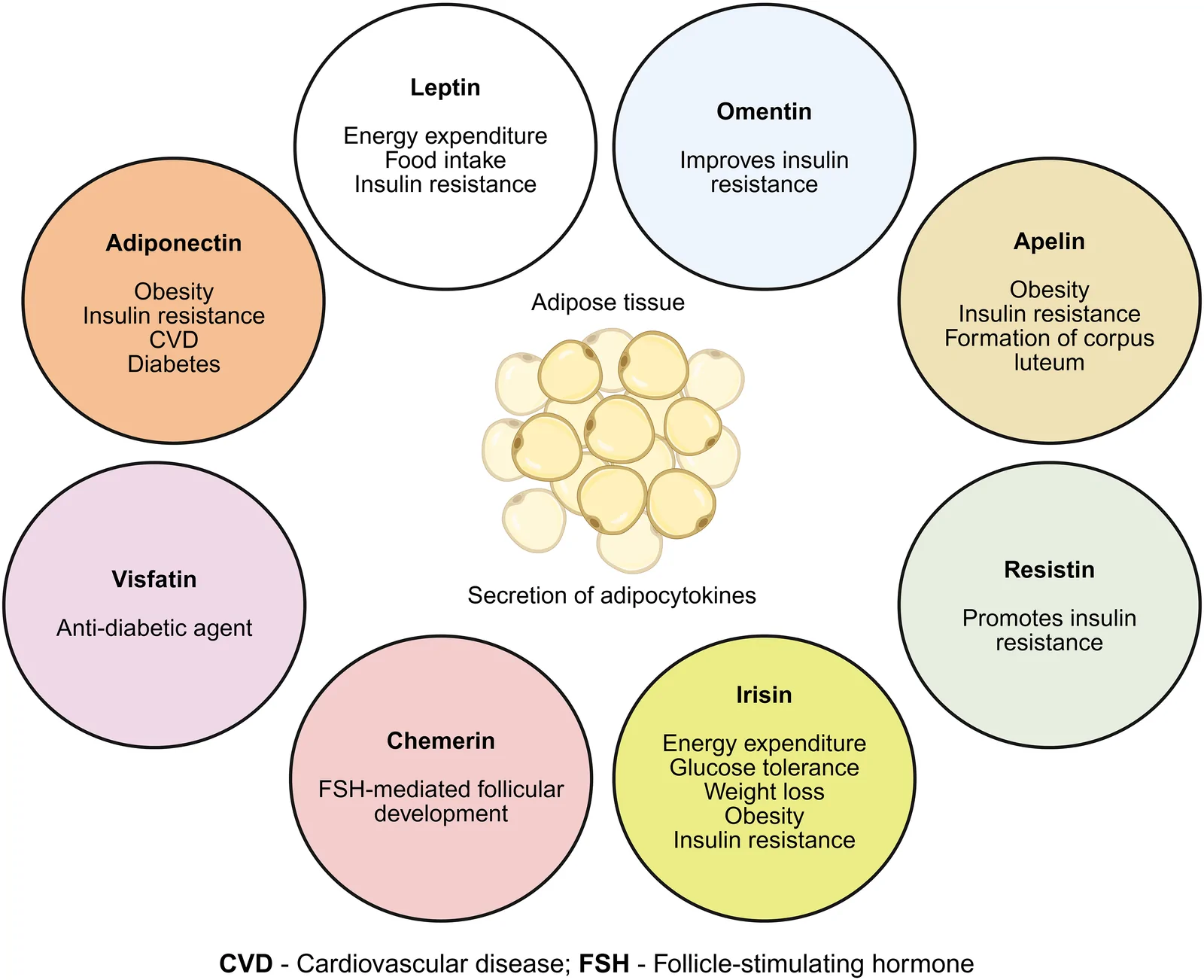
Major adipocytokines secreted by adipose tissue and their roles in obesity, insulin resistance, metabolic dysfunction, and ovarian physiology.

Furthermore, the eventual dysfunction of the adipose tissue contributes to dyslipidemia in PMOS conditions (48). Notably, adipose tissue dysfunction eventually results in the impaired secretion of adipocytokines, leading to dyslipidemia, which is either due to hypo- or hypersecretion of lipids (49). It has been demonstrated that women with PMOS exhibit dyslipidemia, with lower levels of high-density lipoprotein (HDL) and elevated levels of non-HDL lipids such as, low-density lipoprotein (LDL), total cholesterol (TC), and triglycerides (TG) (50) (**Figure 7**). Overall, adipose tissue and its secretory molecules play pivotal roles in PMOS onset.

**Figure 7:**
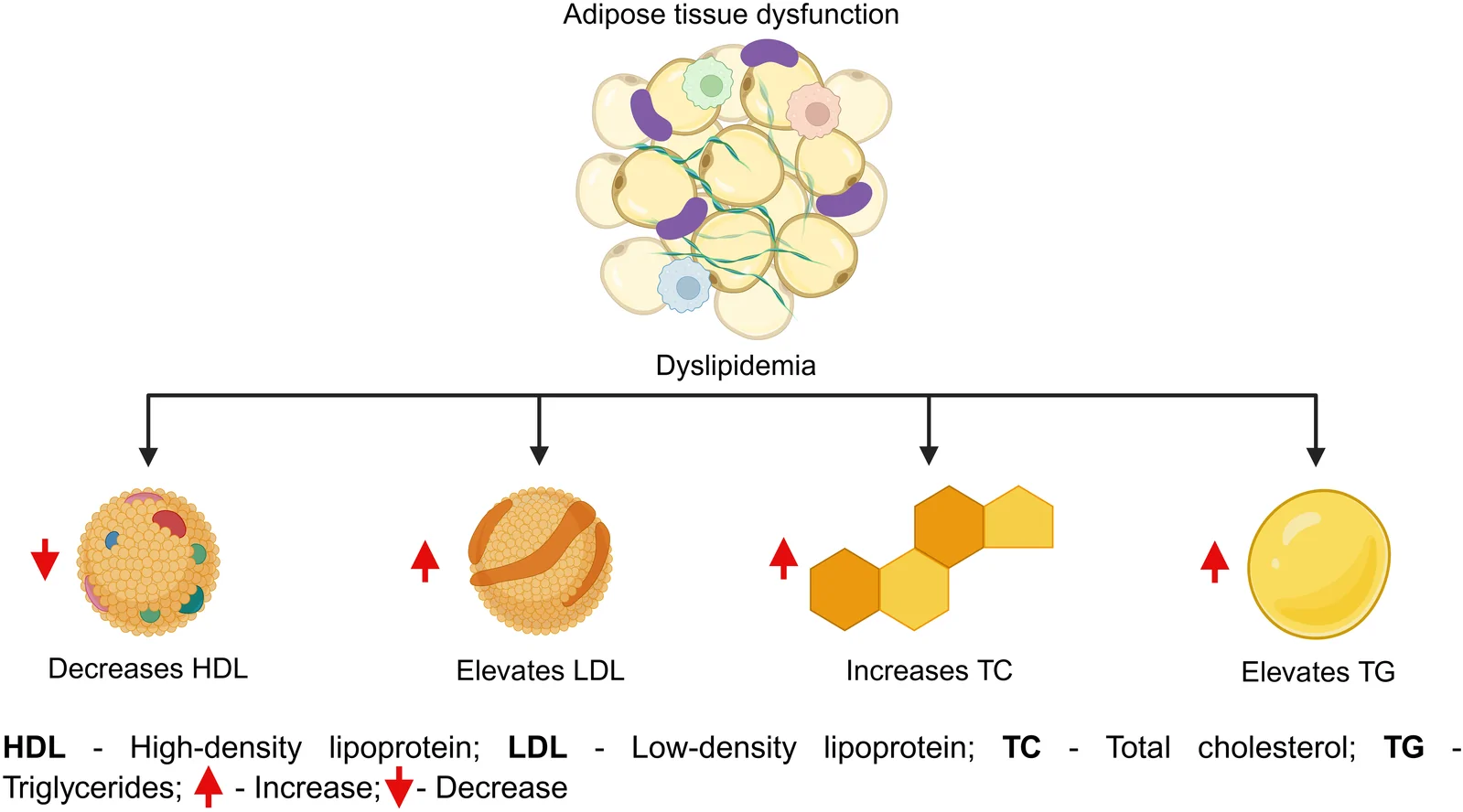
Adipose tissue dysfunction-induced dyslipidemia is characterized by reduced HDL and elevated LDL, total cholesterol, and triglycerides.

Given the complex interplay between endocrine, metabolic, and inflammatory pathways, there is a critical need to develop therapeutic agents that target multiple aspects of PMOS. Here, we highlight the positive effects of diosgenin on various comorbidities of PMOS, thereby exploring the possibility of using diosgenin as a stand-alone therapeutic moiety or in combinatory therapy for the effective management of PMOS.

## 3 Diosgenin and insulin resistance

One of the key metabolic disturbances in PMOS is IR, which occurs due to insults arising from dysregulation of the PI3K/AKT pathway (51). The PI3K/AKT pathway is interlinked with vital regulatory processes, such as cell proliferation, energy metabolism, inflammation, oxidative stress, and the regulation of granulosa cell apoptosis in the ovary. This crucial pathway is disrupted in human and animal PMOS subjects, and restoration of normal PI3K/AKT homeostasis may reduce oxidative stress and apoptosis, thereby improving egg quality (52). The malfunctioning of the PI3K/AKT pathway impairs oocyte health by reducing energy supply (53) due to abnormal insulin signaling. Insulin acts as a precursor molecule for the activation of the PI3K/AKT cascade by binding to the insulin receptor (INSR), which activates the insulin receptor substrate (IRS). Therefore, impairment of the INSR machinery can result in insulin resistance (54), which can further induce obesity, while hyperexpression of INSR contributes to the glucose-lowering effect (55). Furthermore, impaired INSR and insulin resistance may affect the activation and function of glucose transporter type 4 (GLUT4), which is involved in glucose metabolism. These defects were addressed and ameliorated by diosgenin (56) (**Figure 8**). It was found that the obesity-associated insulin resistance could be alleviated by enhancing the expression of *GLUT4, INSR, IRS, PI3K*, and *AKT* (57). In high-fat diet-induced obesity, oxidative stress increases, which in turn promotes the secretion of pro-inflammatory cytokines that suppress *GLUT4* expression and impair glucose homeostasis (58). Uemura et al. (56) demonstrated that supplementation with 2% fenugreek powder in obese diabetic mice and supplementation with diosgenin to 3T3-L1 cells increased the expression of *GLUT4* and *PPARγ*, respectively (59). Additionally, the efficacy of diosgenin in enhancing *GLUT4* expression via the adiponectin receptor 1 (*ADIPOR1*)/Adaptor protein, phosphotyrosine-interacting with PH domain and leucine zipper 1 (*APPL1*) signaling pathway was demonstrated, which improved the function of adiponectin and resulted in the activation of GLUT4-mediated glucose uptake by inhibiting gluconeogenic enzymes (60). Furthermore, diosgenin improves insulin resistance by enhancing the expression of CCAAT/enhancer-binding protein delta (*C/EBPδ*) and *PPARγ* (61). Diosgenin also activates the PI3K/AKT pathway by binding to the estrogen receptor alpha (ERα) and acting as a phytoestrogen (62). In conclusion, the above evidence suggests that IR arising from obesity, impaired PI3K/AKT cascade, dysregulated INSR signaling, and GLUT4 dysfunction can be ameliorated by diosgenin.

**Figure 8:**
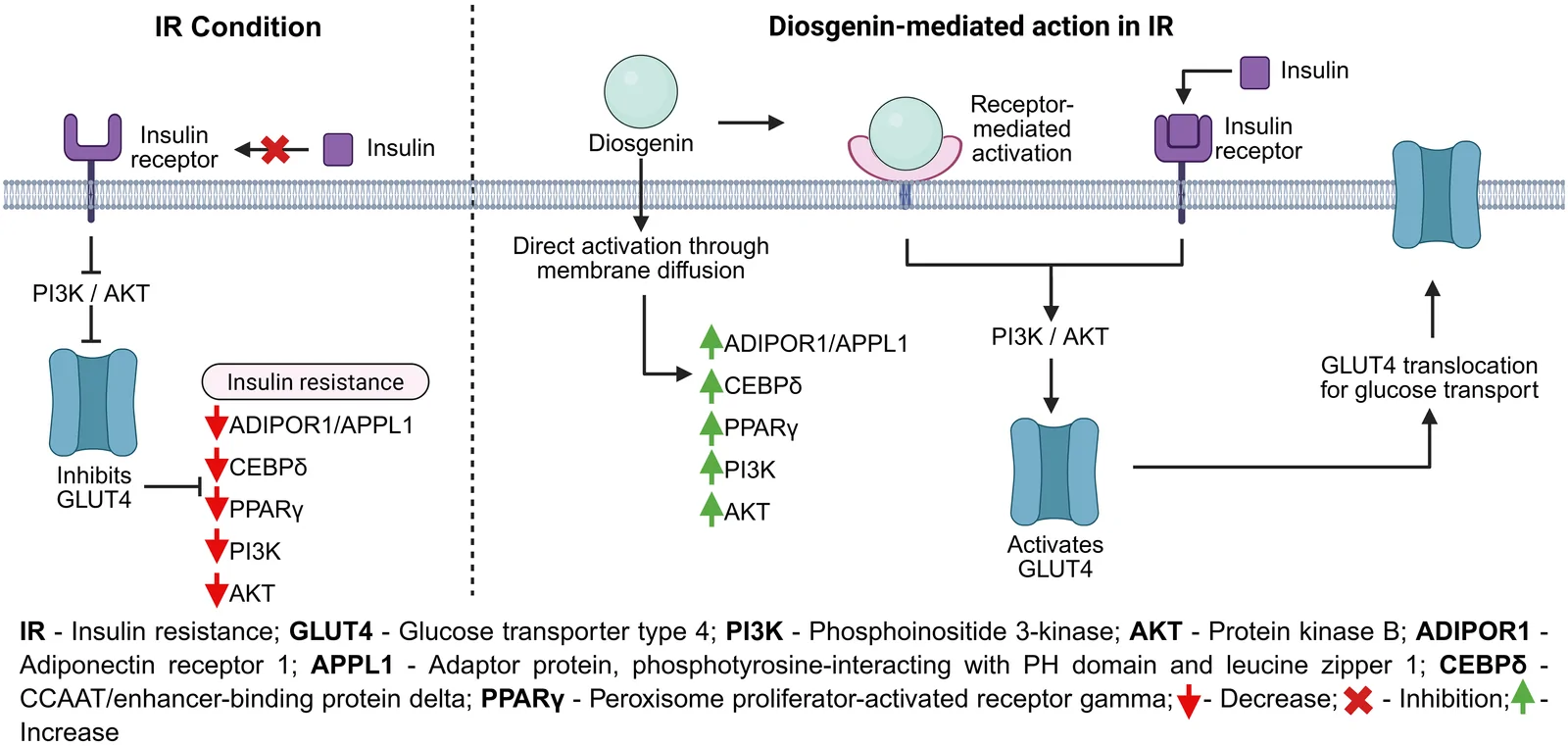
Hypothetical model of diosgenin-mediated improvement of insulin sensitivity and GLUT4-mediated glucose uptake through modulation of ERα and PI3K/AKT signaling pathways in insulin-resistant conditions.

## 4 Diosgenin and chronic inflammation

In addition to metabolic dysfunction, chronic inflammation plays a pivotal role in PMOS, in which immune cells and inflammatory cytokines present in theca cells of the ovary elicit inflammatory responses. Several studies have shown that women with PMOS have higher levels of interleukins (IL)-6, IL-8, TNF-α, IL-1Rα, and IL-17α. These markers are also involved in steroidogenesis, ovarian cell metabolism, proliferation, and follicle maturation (63). It was found that elevated IL-6 levels increase CRP levels, which are positively correlated with PMOS pathogenesis (64, 65). CRP often exacerbates diabetes and cardiovascular defects in patients with PMOS (**Figure 9**) (66). Diosgenin acts as a potent anti-inflammatory agent by inhibiting key inflammatory signaling pathways, including the NF-κB pathway (64). NF-κB acts as a central mediator of inflammatory responses, and its downregulation helps suppress the expression of other pro-inflammatory cytokines, such as TNF-α, IL-1β (67), and IL-6 (68). Another study reported that diosgenin can modulate macrophage polarization and suppress inflammatory signaling pathways by acting on *PPARγ* and NF-κB (28). Therefore, the suppression of chronic inflammation by diosgenin could indirectly improve insulin sensitivity and ameliorate PMOS pathophysiology (64). Overall, there is a high possibility that reducing inflammation-associated defects will restore normal follicular development and ovarian function (69).

**Figure 9:**
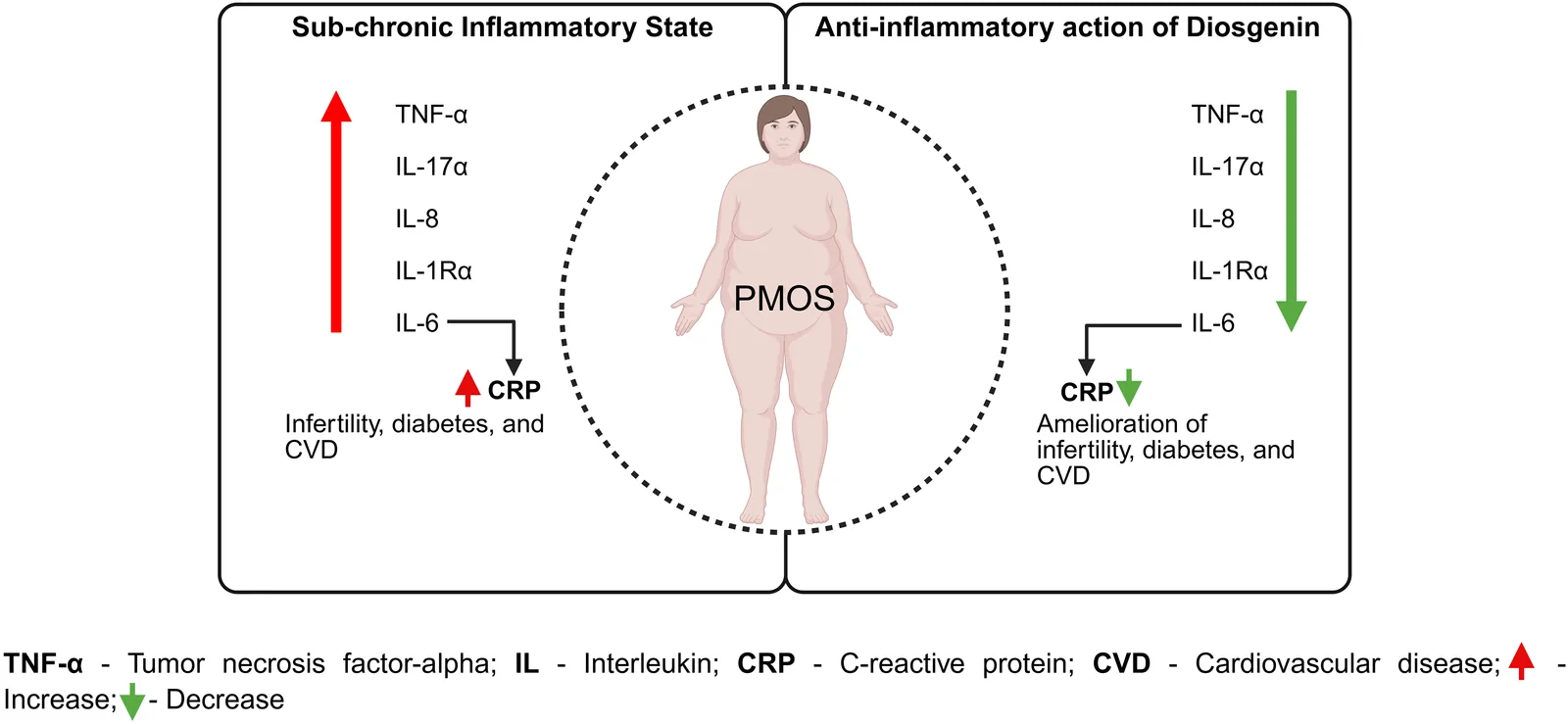
Sub-chronic inflammatory state in PMOS and the anti-inflammatory effects of diosgenin on pro-inflammatory cytokines and associated complications.

## 5 Diosgenin and oxidative stress

Alongside inflammation, oxidative stress contributes to cellular dysfunction in PMOS pathogenesis and other metabolic conditions, as evidenced by alterations in biochemical processes and changes in cellular organelles. A common example of oxidative stress in PMOS is mitochondrial dysfunction (70). Surprisingly, diosgenin supplementation improved mitochondrial membrane potential and alleviated oxidative stress-mediated mitochondrial impairment (68). As shown in **Figure 10**, diosgenin can also reduce oxidative stress by reducing the levels of malondialdehyde (MDA) (64), lipid hydroperoxide (LOOH), and glutathione (GSH), and by improving the levels of superoxide dismutase (SOD), catalase (CAT), glutathione reductase (GR), glutathione peroxidase (GPX), and glutathione-S-transferase (GST) (71). In conclusion, diosgenin is a suitable candidate for ameliorating oxidative stress-associated complications in PMOS.

**Figure 10:**
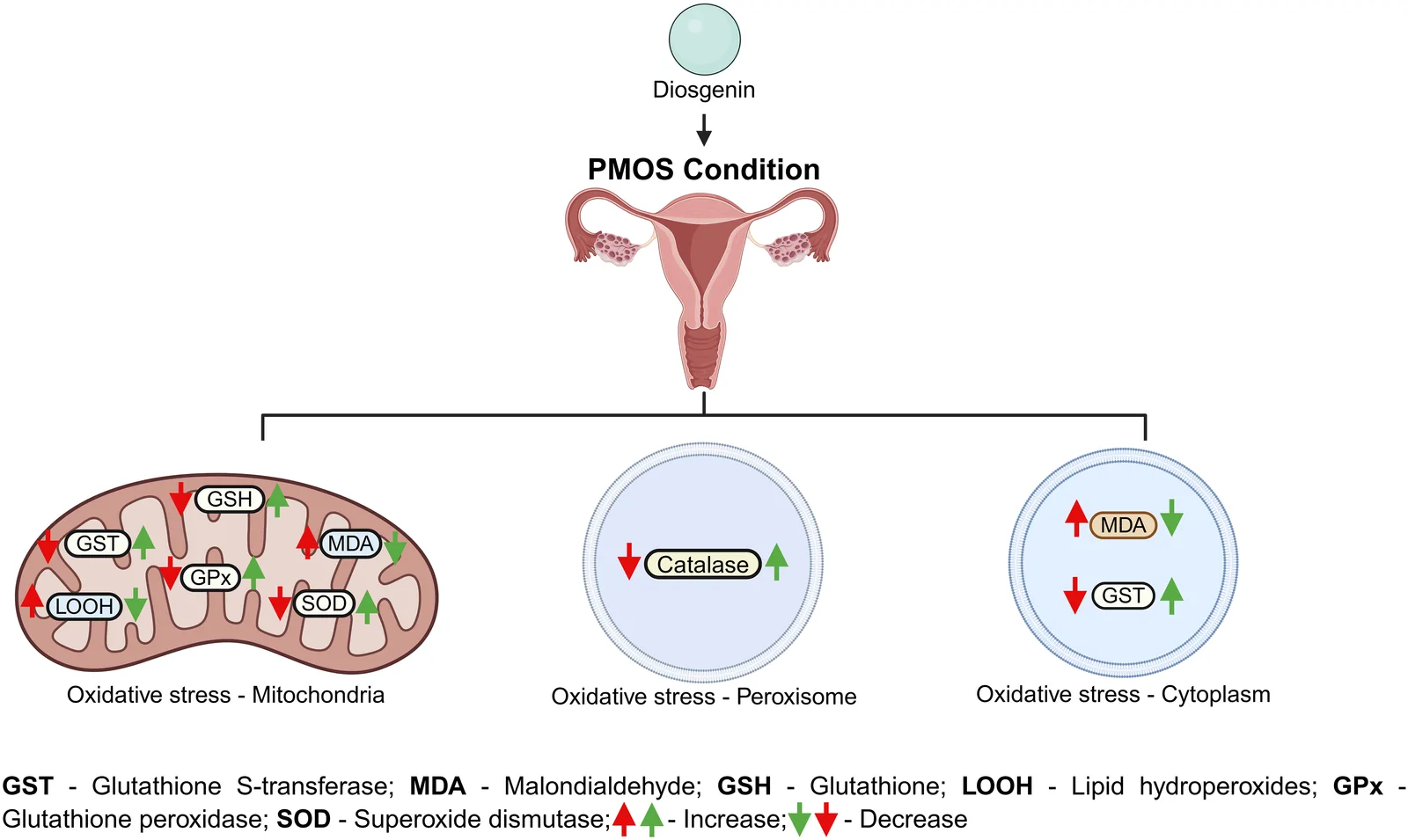
Antioxidant effects of diosgenin against oxidative stress in mitochondrial, cytoplasmic, and peroxisomal compartments under PMOS conditions.

## 6 Diosgenin and lipid metabolism

Metabolic disturbances in PMOS also extend to lipid metabolism, referred to as dyslipidemia. Lipids, such as TC and TG, are negatively linked to the process and quality of egg maturation and development. Conversely, lipids can alter glucose homeostasis and affect reproductive outcomes (72). To date, the exact reason for lipid dysfunction in PMOS is unknown, although IR is known to play a vital role (73). Diosgenin alleviates dyslipidemia by inhibiting the transactivation of liver X receptor alpha (LXRα), thereby reducing hepatic lipid levels. In addition, effective suppression of lipid abnormalities and downregulation of lipogenic gene expression were observed in HepG2 cells supplemented with diosgenin and fenugreek (a diosgenin source). Diosgenin also reduced triglyceride accumulation in high-fat diet (HFD)-induced diabetic mice (74). It has been reported that PMOS patients exhibit elevated LDL levels and reduced HDL levels (75). In one of the studies conducted, lipid complications arising from HFD were resolved with supplementation of diosgenin-coated silver nanoparticles (DIOS-AgNP), resulting in reduced levels of serum cholesterol, LDL, and TG, and improved levels of HDL-cholesterol. Nanoparticle-based delivery of diosgenin has been proposed to enhance its oral bioavailability, improve systemic stability, and facilitate delivery to target tissues, thereby supporting its therapeutic efficacy. Additionally, enhanced expression of antioxidants and normalized levels of plasma lipogenic markers such as, transforming growth factor-1 beta (TGF-β1), sterol regulatory element binding protein 1 c (SREBP1c), acyl-CoA oxidase (ACO) were observed in rats treated with DIOS-AgNPs due to the remarkable reduction in the expression of acetyl-CoA carboxylase (*ACC*), responsible for fatty acid synthesis (76) (**Figure 11**). Therefore, the extensive role of diosgenin in ameliorating lipid-associated comorbidities suggests its therapeutic potential for ameliorating lipid dysfunction associated with PMOS.

**Figure 11:**
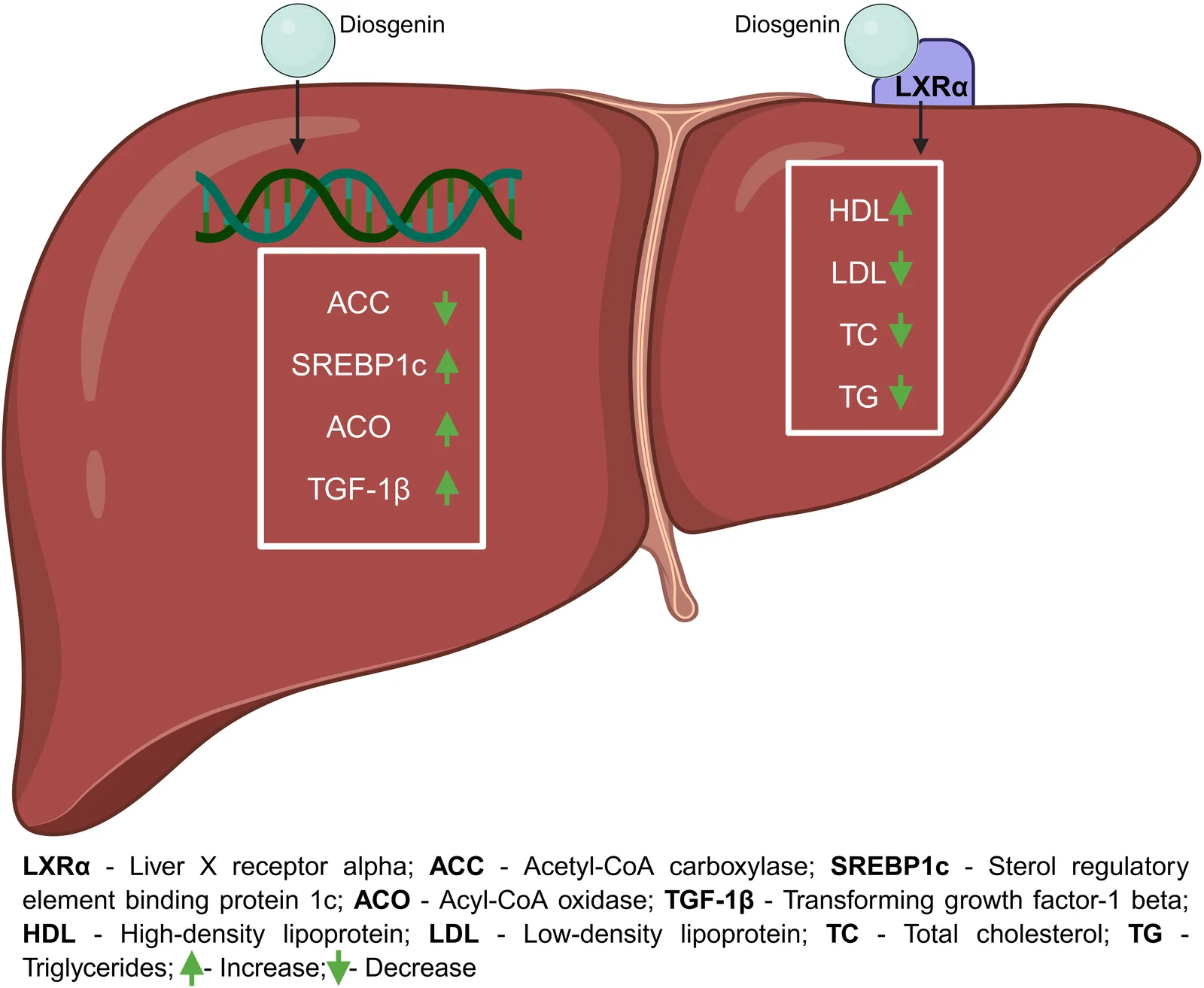
Hypothetical model of (receptor-mediated and membrane diffusion) diosgenin-mediated regulation of hepatic lipid metabolism and improvement of dyslipidemia through the modulation of lipogenic gene expression.

## 7 Diosgenin and hormonal regulation

Despite its metabolic effects, diosgenin may also influence endocrine regulation, particularly ovarian steroidogenesis and endocrine homeostasis. A study used rhizome powder of *Dioscorea* spp. as a source of diosgenin, which has been proven to elevate the expression of steroidogenic proteins such as steroidogenic acute regulatory protein (StAR), CYP19A1, and estrogen receptor beta (ERβ) in the rat ovarian granulosa cells (77) (**Figure 12**). These markers are crucial in regulating steroidogenesis and maintaining ovarian health (78). Following that, it was found that diosgenin treatment reduced the release of progesterone and stimulated the conversion of testosterone in the porcine ovarian granulosa cells, whereas in rabbit ovarian fragments, diosgenin elevated the release of progesterone and insulin-like growth factor 1 (IGF-1) (23). This reveals the species-specific effects of diosgenin on progesterone secretion and modulation of steroidogenesis. Put together, diosgenin has the potential to facilitate hormone secretion at the molecular level. Administering the *Ficus religiosa* extract to the letrozole-induced PMOS rodent model led to elevated expression of *PPARγ* and increased levels of *CYP19A1* (13). At this point, targeting *PPARγ* with diosgenin will provide valuable insights into the mechanistic activation of aromatase to alleviate the complex endocrinopathy. Put together, a comprehensive study on the effect of diosgenin in endocrine regulation is highly warranted. We suggest that diosgenin could modulate *PPARγ* and thereby it can activate *CYP19A1*; however, experimental evidence is yet to be established. **Table 1** provides a summary of the evidence reviewed in this article.

**Figure 12:**
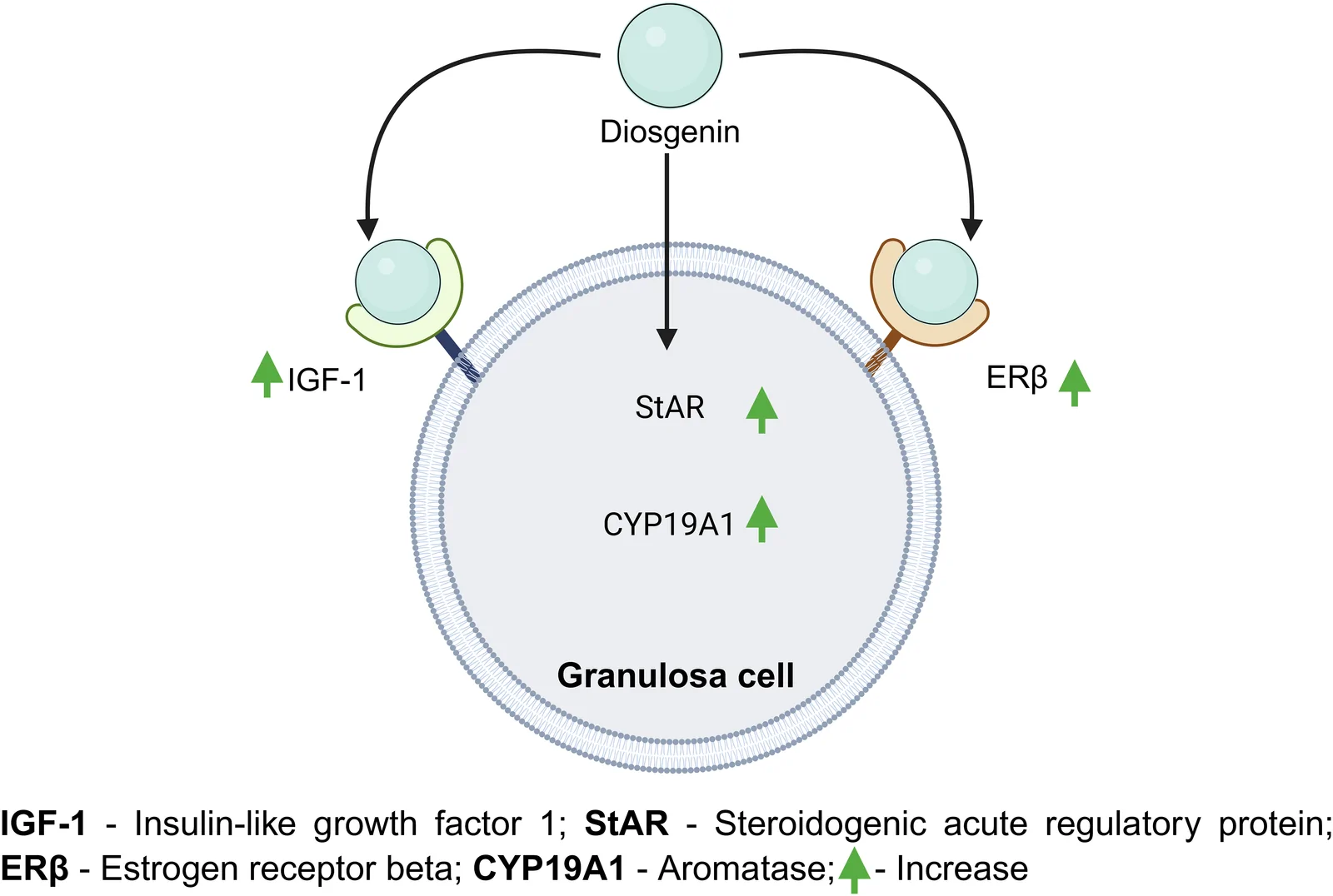
Diosgenin-mediated modulation of granulosa cell steroidogenesis through regulation of IGF-1, ERβ, StAR, and CYP19A1 expression.

**Table 1:** Summary of the therapeutic effects of diosgenin on PMOS-associated pathological pathways.

PMOS feature	Experimental model	Major target/pathway	Diosgenin action	Key outcome	Limitation	Reference
Insulin resistance	HFD mice and HFD rats	PI3K/AKT, GLUT4, and INSR	↑ PI3K, ↑ AKT, ↑ GLUT4	Improved glucose uptake	Non-ovarian models	(57–59)
Inflammation	HFD mice and Macrophages	NF- κB, TNF-α, and IL-6	↓ NF-κB activation	Reduced inflammatory cytokines	Not validated in PMOS	(28, 64)
Oxidative stress	Diabetic rats	SOD, CAT, and GPx	↑ Antioxidant enzymes	Improved mitochondrial function	Diabetic model	(68, 71)
Dyslipidemia	HFD mice	LXRα and ACC	↓ TG, ↓ LDL, ↑ HDL	Improved lipid metabolism	Not specific to PMOS	(74, 77)
Steroidogenesis	Rabbit ovarian fragments and Porcine granulosa cells	Progesterone and IGF-1	Species-dependent	Contradictory progesterone regulation	Unknown mechanism	(23)
PPARγ expression	Computational study, 3T3-L1 cells, and HFD mice	PPARγ	Exhibits effective binding in computational study, upregulation in 3T3-L1 cells and downregulation in mice model	Context-dependent	Contradictory evidence	(83, 84)

## 8 Evidence of diosgenin on the modulation of *PPARγ*

Given the emerging role of PPARγ in both metabolic and reproductive regulation, understanding its interaction with diosgenin is critical. *PPARγ*, a ligand-activated transcription factor, is involved in vital regulatory mechanisms (79). *PPARγ* is responsible for the synthesis of steroid hormones in granulosa cells of the ovary, and its altered expression leads to subfertility (80). A recent study demonstrated that siRNA-mediated knockdown of *PPARγ* inhibits granulosa cell proliferation and estrogen biosynthesis. Additionally, *PPARγ* silencing significantly increased the expression of apoptosis markers and decreased the expression of cell cycle regulators (81). In this regard, the activation of *PPARγ* is pivotal for the regulation of early ovarian development (82). Therefore, restoring impaired *PPARγ* expression or signaling may be a crucial factor in PMOS.

A computational study demonstrated that diosgenin exhibited the highest binding affinity (-6.73) to PPARγ at its active site. Furthermore, diosgenin increased *PPARγ* expression in 3T3-L1-differentiated preadipocytes (83). In contrast, HFD increases the expression of *PPARγ*, indicating it as a marker of lipogenesis. This hyperactivation of *PPARγ* was suppressed by diosgenin by modulating ERβ, thereby preventing the formation of PPARγ-RXR dimers. Moreover, this inhibitory machinery, regulated by diosgenin, had normalized *PPARγ* expression levels (84). Though the results of both studies were contradictory, it was beneficial in terms of context-specific modalities. At the molecular level, diosgenin alters *PPARγ* expression, alleviates adipose-associated complications and endoplasmic reticulum stress, and improves antioxidant levels in the pancreas (85) (**Figure 13**). Though the empirical evidence provided in **Table 2** suggests the possible role of diosgenin on *PPARγ*, a holistic molecular study is warranted to unravel the potential of diosgenin in the management of PMOS.

**Figure 13:**
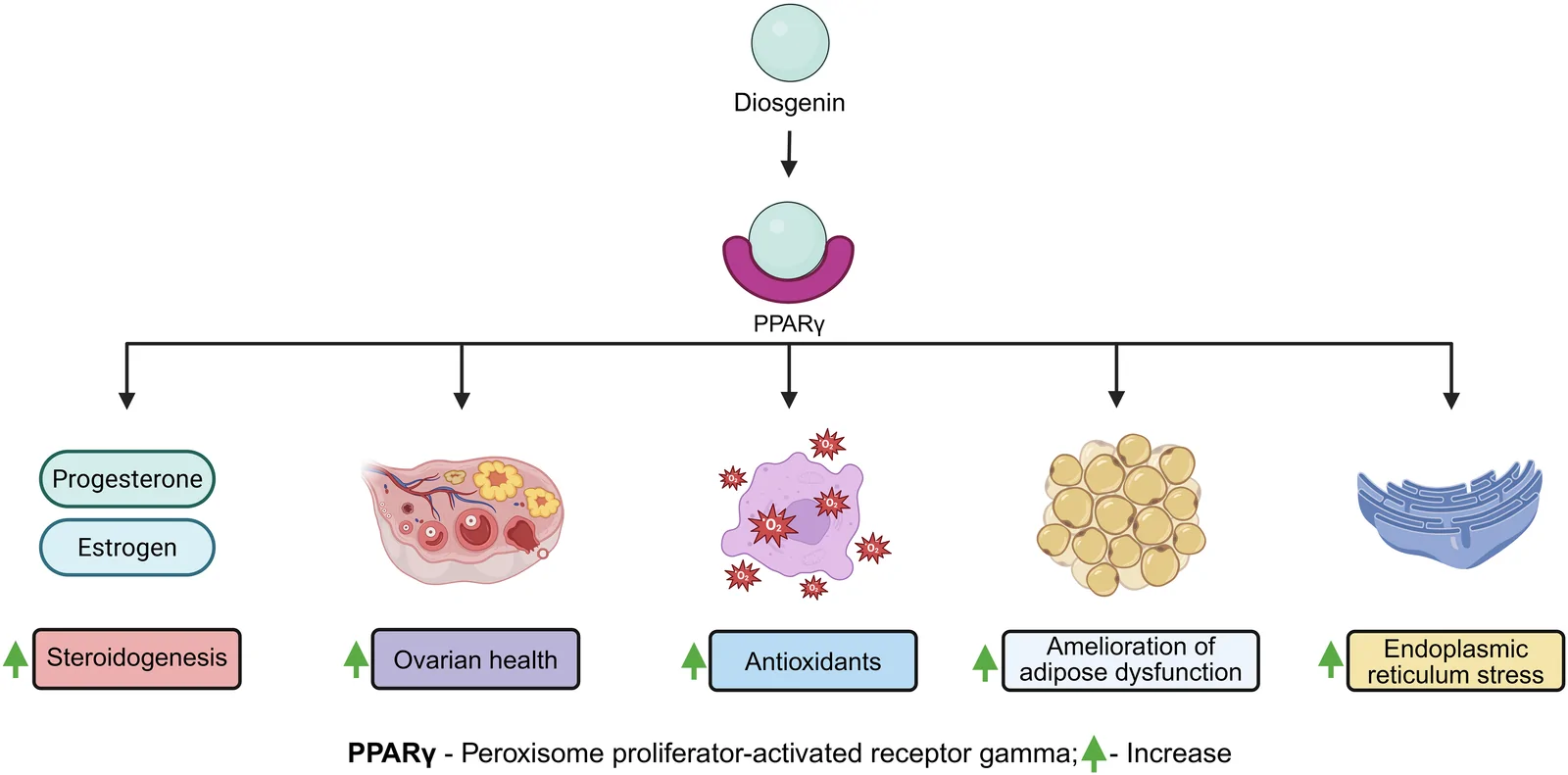
Therapeutic effects of diosgenin mediated through PPARγ activation on steroidogenesis, ovarian function, antioxidant defense, adipose dysfunction, and endoplasmic reticulum stress in PMOS.

**Table 2:** Current evidence supporting PPARγ as the primary therapeutic target of diosgenin in PMOS.

Model	Effect on PPARγ	Downstream target	Conclusion	Limitation	Reference
Computational study and 3T3-L1 cell line	↑ PPARγ	Glucose metabolism	Supports PPARγ activation	Non-ovarian origin	(83)
HFD rats	↓ PPARγ	ERβ	Prevented lipogenesis	Metabolic model	(84)
Letrozole PMOS rats	↑ PPARγ	↑ CYP19A1	Improved steroidogenesis	No diosgenin administration	(13)
Macrophages	PPARγ and NF-κB	Anti-inflammatory	Reduced inflammation	Immune cells	(28)

## 9 Pre-clinical and clinical evidence

An in-silico study suggests that diosgenin may interfere with the formation of the follicle, hormonal regulation, and estrogen signaling by exhibiting effective binding affinity with ESR1 (-8.9 kcal/mol), AKT1 (-8.8 kcal/mol), TNF-α (-10.8 kcal/mol), and PPARγ (-9.3 kcal/mol) (86). *In vitro* studies have demonstrated that diosgenin improves insulin signaling, reduces oxidative stress, and regulates inflammatory mechanisms in various metabolic systems (87). Similarly, studies in ageing mouse models showed that diosgenin improves ovarian reserve in mice (88). Despite these novel preclinical findings, clinical evidence in humans remains limited. A recent study by Russo et al. (89) showed that combining diosgenin with other supplements, such as vitamin D and α-lactalbumin, improved menstrual cyclicity, ameliorated hyperandrogenism, and altered the LH/FSH ratio in PMOS phenotype D patients (89). All these evidence from in silico, *in vitro*, and *in vivo* studies supports the therapeutic efficacy of diosgenin in managing PMOS and the associated metabolic complications (**Table 3**).

**Table 3:** Current translational evidence supporting the therapeutic potential of diosgenin in PMOS.

Evidence level	Experimental model	Major findings	Clinical significance	Reference
In silico	Computational studies	PPARγ, AKT1, and ESR1 binding	Hypothesis generation	(83, 86)
In vitro	3T3-L1 cells, macrophages, and granulosa cells	Improved signaling	Mechanistic evidence	(23, 28, 59, 83)
In vivo	HFD mice, diabetic rats, and PMOS rats	Metabolic improvement	Preclinical support	(13, 57, 60, 64, 71, 74, 77, 84, 85)
Clinical	Phenotype D PMOS patients	Improved menstrual cyclicity and LH/FSH ratio	Limited evidence, and focuses on PMOS Phenotype D	(89)

## 10 Discussion

The curated evidence across the preceding sections identifies diosgenin as a multi-target phytotherapeutic moiety for ameliorating PMOS pathophysiology by alleviating insulin resistance, low-grade chronic inflammation, oxidative stress, dyslipidemia, and hormonal dysregulation. However, the lack of mechanistic evidence, along with several limitations, weakens the strength of the proposed therapeutic proposition.

The existing literature presents unresolved inconsistencies that hinder understanding of the biological effects of diosgenin. The primary limitation is the absence of ovarian-tissue-specific validation of diosgenin as a stand-alone therapeutic molecule. Although diosgenin has consistently exhibited beneficial effects in metabolic, inflammatory, oxidative stress, and steroidogenic pathways, most of the evidence presented is derived mainly from non-ovarian experimental systems or from metabolic rodent models. Also, effect of diosgenin on oocyte quality and folliculogenesis remains elusive and requires direct experimental validation. Furthermore, it remains unclear whether diosgenin alone is sufficient to restore endocrine and metabolic anomalies, or whether its therapeutic efficacy would be improved when used as a supplement, warranting holistic evidence.

The biological effects of diosgenin appear to be highly context-dependent, with studies reporting both upregulation (83) and suppression of *PPARγ* expression (84) across different experimental systems and showing opposing effects on progesterone secretion in granulosa cells of porcine and rabbit (23). The exact pathway underlying this effect remains unknown; however, the study suggested that ovarian granulosa cells might be the primary target of diosgenin. Furthermore, diosgenin increased progesterone secretion in rabbit ovarian fragments but reduced progesterone production in porcine granulosa cells. It was proposed that the reduced progesterone levels observed in the porcine cells may result in the elevated downstream conversion of steroid hormones (testosterone and estrogen) (23). However, this was not experimentally validated and remains hypothetical. This contradiction may reflect species-specific differences, experimental conditions, dosage, treatment duration, or context-dependent regulation of *PPARγ* signaling. Therefore, the reported evidence is descriptive rather than mechanistic, leaving an unresolved clinical question about whether diosgenin can restore or worsen the hormonal imbalance in the hyperandrogenic phenotype. This concern is amplified by reports stating that diosgenin-induced testosterone stimulation in porcine models warrants holistic evidence to prove the clinical efficacy of diosgenin in the treatment of PMOS subjects.

Furthermore, a rising concern is due to the translational gap created by computational and preclinical evidence rather than clinical validation. Molecular docking studies explicitly predict the favorable binding interaction between diosgenin and receptors, such as ESR1, AKT1, TNF-α, and PPARγ (86). The findings established a hypothetical conclusion, whereas experimental evidence for functional bioactivity, tissue penetration, and downstream signaling *in vivo* remains limited. Moreover, the poor water solubility and low oral bioavailability of diosgenin limit its therapeutic translation, as these pharmacokinetic barriers may reduce systemic exposure and targeted delivery. This could be addressed by encapsulating diosgenin in nanoparticles to improve its solubility, oral bioavailability, and pharmacological efficacy (76) in preclinical models, but its application in PMOS remains poorly explored. Likewise, a single pilot-scale human study evaluated the combinatorial effects of diosgenin with vitamin D and α-lactalbumin in PMOS phenotype D patients, which is insufficient to confirm the therapeutic potential of diosgenin (89). Additionally, this study observed improvements only in menstrual cyclicity and the LH/FSH ratio. Still, the lack of mechanistic evidence raises additional translational research questions, and the normalization of other hallmarks of PMOS in the left-out phenotypes remains unexplored. Also, the study does not explain the comparative effect of combination therapy with reference to standard drugs such as metformin and pioglitazone. Furthermore, no clinical study has evaluated diosgenin as a stand-alone treatment option, making it difficult to distinguish its independent therapeutic effects from those of combination therapy.

Lastly, the elucidated mechanistic pathways of diosgenin remain fragmentary and encompass metabolic, inflammatory, oxidative, lipidomic, and steroidogenic pathways. However, these pathways remain discrete rather than being the central component of an integrated signaling network. However, the biological relevance of signaling hubs, including PPARγ and TNF-α-mediated IRS-1 phosphorylation, remains elusive, thereby limiting its ability to explain the molecular crosstalk between insulin resistance and inflammatory pathways. Furthermore, the clinical efficacy of diosgenin remains questionable due to the absence of a system-level framework. This leaves it unclear whether the observed effects were driven by a single primary mechanism or by the modulation of multiple interconnected pathways.

Altogether, the presented observations indicate that diosgenin is biologically plausible and targets multiple pathways relevant to PMOS pathophysiology. The published findings are predominantly computational and preclinical, largely non-ovarian in origin, and exhibit heterogeneity across experimental systems. These limitations do not weaken the biological rationale for diosgenin as a therapeutic candidate. Instead, they define the critical research gaps that need to be addressed to make diosgenin a clinically viable therapeutic candidate to enable successful clinical translational research.

## 11 Conclusion and future perspectives

PMOS is a complex, multifactorial endocrine metabolic syndrome affecting women of reproductive age, which is characterized by hormonal imbalance and metabolic dysfunctions. The clinical manifestations of PMOS includes hyperandrogenism, irregular menstruation, and multiple cystic ovarian morphology. To date, the medications prescribed for PMOS are synthetic PPARγ agonists, such as thiazolidinediones and synthetic insulin-sensitizing agents such as metformin to alleviate metabolic-associated complications, which have adverse side effects. As a result, interest in alternative phytotherapy approaches has increased significantly. Among the diverse phyto-substitutes, one promising molecule is diosgenin, a steroidal sapogenin present in *Dioscoreae* spp. Emerging evidence suggests that diosgenin may modulate oxidative stress, lipid metabolism, hormonal dysfunction, and other associated pathological features of PMOS.

Furthermore, involvement of diosgenin in the regulation of *PPARγ,* PI3K/AKT signaling, steroidogenesis, and ovarian-adipose crosstalk may provide deeper insights into its pharmacological applications. Collectively, the existing evidence highlights the therapeutic promise of diosgenin and its potential to be prescribed as a stand-alone phytotherapeutic agent; however, several important research gaps in the regulation of steroidogenesis remain, warranting mechanistic evidence. Further, few studies have demonstrated the regulatory role of diosgenin on *PPARγ*, yet studies on the modulation of *CYP19A1* are limited. As discussed above, *PPARγ* acts as a regulator of aromatase, and whether altering *PPARγ* via diosgenin can restore the hormonal imbalance remains unclear. For this, clinical trials such as randomized controlled trials (RCTs) and placebo-controlled trials (PCTs) are required to validate the safety profile and therapeutic efficacy of diosgenin for human consumption. Overall, addressing these approaches would help in the development of diosgenin as a viable alternative treatment strategy for PMOS and associated metabolic conditions. However, further clinical validations are required to establish its efficacy and facilitate its translation into clinical practice.
